# Conservation of the genes for HC-toxin biosynthesis in *Alternaria jesenskae*

**DOI:** 10.1186/1471-2180-13-165

**Published:** 2013-07-17

**Authors:** Wanessa D Wight, Roman Labuda, Jonathan D Walton

**Affiliations:** 1 Department of Energy Plant Research Laboratory, Michigan State University, 612 Wilson Road, Room 210, East Lansing, MI 48824, USA; 2 Romer Labs Division Holding GmbH, Technopark 1, Tulln 3430, Austria; 3 Current address: 4351 E. Strange Hwy, Grand Ledge, MI 48837, USA

**Keywords:** Nonribosomal peptide synthetase, Host-selective toxin, Plant pathogenesis, Cyclic peptide

## Abstract

**Background:**

HC-toxin, a cyclic tetrapeptide, is a virulence determinant for the plant pathogenic fungus *Cochliobolus carbonum*. It was recently discovered that another fungus, *Alternaria jesenskae*, also produces HC-toxin.

**Results:**

The major genes (collectively known as *AjTOX2*) involved in the biosynthesis of HC-toxin were identified from *A. jesenskae* by genomic sequencing. The encoded orthologous proteins share 75-85% amino acid identity, and the genes for HC-toxin biosynthesis are duplicated in both fungi. The genomic organization of the genes in the two fungi show a similar but not identical partial clustering arrangement. A set of representative housekeeping proteins show a similar high level of amino acid identity between *C. carbonum* and *A. jesenskae*, which is consistent with the close relatedness of these two genera within the family Pleosporaceae (Dothideomycetes).

**Conclusions:**

This is the first report that the plant virulence factor HC-toxin is made by an organism other than *C. carbonum.* The genes may have moved by horizontal transfer between the two species, but it cannot be excluded that they were present in a common ancestor and lost from other species of *Alternaria* and *Cochliobolus*.

## Background

Many secondary metabolites play important ecological roles in the interactions between microbes and other organisms. Some, such as the host-selective toxins, are virulence factors for plant pathogenic fungi [1]. Two genera, *Cochliobolus* and *Alternaria*, both in the Pleosporaceae of the Dothideomycetes, have particularly exploited this strategy to increase their pathogenic fitness and to extend their host range to new species and strains of crop plants ranging from cereals (maize, oats) to dicotyledonous plants (strawberry, citrus, tobacco, tomato) [2-4].

HC-toxin is a cyclic tetrapeptide of structure cyclo(D-Pro-L-Ala-D-Ala-L-Aeo), where Aeo stands for 2-amino-9,10-epoxi-8-oxo-decanoic acid. HC-toxin is a host-selective toxin that endows the pathogenic fungus *Cochliobolus carbonum* with exceptional virulence on maize varieties that lack a functional copy of *HM1* and/or *HM2*, both of which encode a carbonyl reductase that detoxifies HC-toxin [5]. A minority of natural isolates of *C. carbonum*, designated race 1, make HC-toxin [6]. Only maize lines of genotype *hm1*/*hm1, hm2*/*hm2* are sensitive to HC-toxin and hence susceptible to race 1 isolates of *C. carbonum*. Because all grasses have functional orthologs of *HM1*, HC-toxin-producing pathogens (not necessarily *C. carbonum*) have apparently exerted significant selective pressure on plants in the Poaceae throughout their evolutionary history [7].

The central enzyme in HC-toxin biosynthesis, HTS1, is a four-module nonribosomal peptide synthetase (NRPS) containing one epimerase domain [5]. Other known genes involved in HC-toxin biosynthesis include *TOXA*, encoding a member of the major facilitator superfamily of transporters; *TOXC*, encoding a fatty acid synthase beta subunit; *TOXE*, encoding a pathway-specific transcription factor; *TOXF*, encoding a putative branched chain amino acid aminotransferase; and *TOXG*, encoding an alanine racemase. A seventh gene found in the *TOX2* locus, *TOXD*, encodes a predicted short-chain alcohol dehydrogenase, but its disruption gave no phenotype in HC-toxin production or virulence [5].

The genes involved in HC-toxin biosynthesis, called collectively *TOX2*, are organized into a diffuse cluster that spans >500 kb. All of the known genes are duplicated or triplicated within this region, with some variation in copy number and chromosomal location among different race 1 strains [8,9] . Natural toxin-nonproducing isolates of *C. carbonum* (designated race 2) completely lack all of the known biosynthetic genes [5,8]. The *TOX2* locus is meiotically unstable [10].

HC-toxin is an inhibitor of histone deacetylases (HDACs) of the RPD3 class [11,12]. A chemically related HDAC inhibitor, apicidin, is made by *Fusarium incarnatum* (=*F. semitectum*) [13]. Like HC-toxin, apicidin is a cyclic tetrapeptide containing a D-imino acid and an L-amino acid with an aliphatic R-group (Aeo in the case of HC-toxin and 2-amino-8-oxo-decanoic acid in the case of apicidin). The gene cluster responsible for apicidin biosynthesis has been characterized, and many of the genes of the apicidin gene cluster have as their closest known homologs the genes of *TOX2*, including *HTS1*, *TOXA*, *TOXE*, and *TOXF*[14].

During a screen for new HDAC inhibitors, a new species of *Alternaria* (*A. jesenskae*) that produces HC-toxin was discovered [15]. *A. jesenskae* was isolated from seeds of *Fumana procumbens*, a shrubby perennial with a wide geographic distribution, but it is not known if *A. jesenskae* is pathogenic. A situation in which two fungi in different genera produce the same compound is unusual and presents an opportunity to explore the evolution of a complex secondary metabolite, especially one with a strong evolutionary impact on the cereals. Here we document the identification and characterization of the genes for HC-toxin biosynthesis in *A. jesenskae*.

## Results

### *Alternaria jesenskae* produces HC-toxin

**Figure 1:**
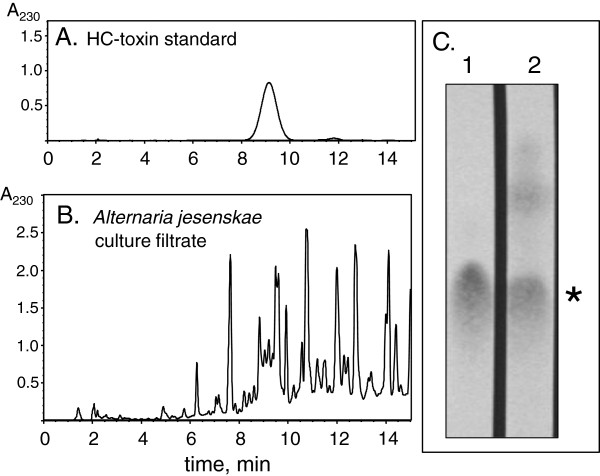
**Analysis of HC-toxin from *A. jesenskae *by HPLC and TLC. (A)** HPLC of standard HC-toxin (10 μg). **(B)** HPLC of *A. jesenskae* culture filtrate extracted with dichloromethane (400 μl equivalent crude culture filtrate). Detection in both cases was at 230 nm. **(C)** TLC of (1) native HC-toxin, and (2) material from *A. jesenskae* eluting between 8 and 10 min from HPLC of the separation shown in panel B. Visualization used an epoxide-specific reagent [45]. The asterisk indicates the position of HC-toxin.

An isolate of *A. jesenskae* was obtained and its taxonomic identity confirmed by sequencing of the ITS regions [15]. Culture filtrates of *A. jesenskae* were fractionated by reverse phase HPLC. No particular peak was seen at the retention time of HC-toxin (Figure 1A), but fractions with the same retention time as native HC-toxin contained an epoxide-containing compound with the same R_f_ on TLC as HC-toxin (Figure 1B). The mass of this compound was determined to be 437.2407 ± 0.0007 ([M + H]^+^), compared to a calculated mass of 437.2400 for a compound with the elemental composition of HC-toxin (C_21_H_32_N_4_O_6_) [16]. These results confirm the observation that *A. jesenskae* makes HC-toxin.

### *Alternaria jesenskae* has unmistakable orthologs of the *TOX2* genes

**Figure 2:**
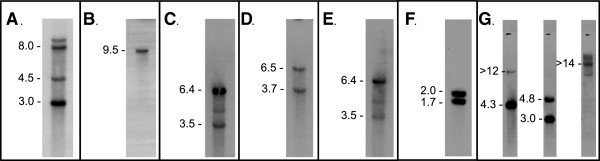
**Southern blots of the genes of *AjTOX2*.** In every case, the DNA was cut with enzymes that did not cut within the probes. DNA was cut with: **(A)** BamHI, and probed with *AjTOXA*; **(B)** NheI, and probed with *AjTOXC*; **(C)** AatII, BclI, and KpnI, and probed with *AjTOXD*; **(D)** NruI and EcoRI, and probed with *AjTOXE*; **(E)** AatII, BclI, and KpnI, and probed with *AjTOXF*; **(F)** AtII, BclI, and KpnI, and probed with *AjTOXG*; **(G)** (left to right), NaeI, EagI, or AatII, and all three probed with *AjHTS1*. See Additional file 1: Table S2 for the PCR primers used to amplify the probes used on the Southern blots.

The genome of *A. jesenskae* was determined to ~10× coverage by pyrosequencing followed by assembly. Using BLASTN and TBLASTN, strongly related sequences of each of the known seven *TOX2* genes from *C. carbonum* were found in the genome of *A. jesenskae* (Table 1). These will be collectively referred to as *AjTOX2*. Southern blotting of *A. jesenskae* DNA cut with diagnostic restriction enzymes indicated that all of the seven known *TOX2* genes except perhaps *AjTOXC* were present in at least two copies in the genome of *A. jesenskae* (Figure 2), as they are in *C. carbonum*[9].

**Table 1:** **Percent identities at the DNA (nucleotide) and protein (amino acid) levels for the *TOX2 *genes of *C. carbonum *and the *AjTOX2 *genes of *A. jesenskae***

**Gene**	***C. carbonum***		***A. jesenskae***	
	**DNA**	**protein**	**DNA**	**protein**
*HTS1*^a^	85	82		
*TOXA* (1)	81	80	95	95
*TOXA* (2)	82	80		
*TOXC*	83	80		
*TOXD* (1)	85	81	95	93
*TOXD* (2)	86	82		
*TOXE* (1)	74	64	85	76
*TOXE* (2)	72	58		
*TOXF* (1)	84	84	97	94
*TOXF* (2)	85	85		
*TOXG* (1)	80	81	92	93
*TOXG* (2)	81	82		

The values in the columns headed “*C. carbonum*” are the identities between the genes and proteins of *C. carbonum* and *A. jesenskae*, and the values in the columns headed “*A. jesenskae*” are the identities between the two copies within *A. jesenskae*, when relevant.

^a^Because of the impossibility of resolving both copies of *HTS1* in *C. carbonum*, only one copy is included.

### The HC-toxin biosynthetic genes of *A. jesenskae*

#### *AjHTS1*- Non-ribosomal peptide synthetase (NRPS)

HC-toxin synthetase (HTS1) in *C. carbonum* is a 5218-amino acid NRPS encoded by a 15.7-kb open reading frame. It contains four modules with one epimerase domain between modules 1 and 2 [17,18]. BLASTN and TBLASTN indicated numerous and overlapping contigs related to *HTS1* in *A. jesenskae*. Following computational and manual assembly, the complete sequence of *AjHTS1* was deduced. The encoded NRPS contains 5207 amino acids, four modules, and an epimerase domain between modules 1 and 2. *A. jesenskae* contains at least two copies of *AjHTS1*, but it was not always possible to deduce which contig was derived from which copy. *AjHTS1* and *HTS1* of *C. carbonum* share 84% (nucleotide) and 82% (amino acid) identity (Table 1), which is higher than either one to any other NRPS in the GenBank or JGI databases. Like *HTS1*, *AjHTS1* has no predicted introns.

#### *AjTOXA* – major facilitator superfamily (MFS) transporter

**Figure 3:**
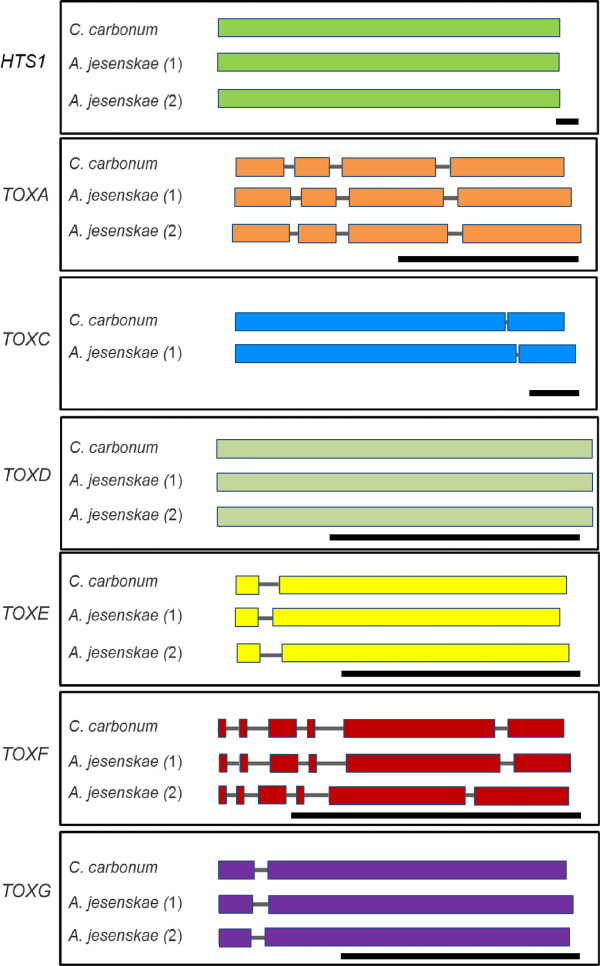
**Intron/exon structures of *C. carbonum *and *A. jesenskae TOX2 *genes.** All structures were experimentally determined by comparison of cDNA sequences with genomic sequences. The numbers in parantheses indicate the multiple copies of each in gene in *A. jesenskae.* The black bars in the lower right corner of each box indicate 1 kb.

Both BLASTN and TBLASTN identified at least six contigs, some overlapping, with strong homology to *TOXA* of *C. carbonum*. The *AjTOXA* contigs were manually aligned and assembled, revealing that *A. jesenskae* has at least two copies of *TOXA (AjTOXA-1* and *AjTOXA-2)*, although by Southern blotting there may be as many as four (Figure 2). Using AjTOXA as the search query against the GenBank and JGI databases, TOXA gave the strongest hit (79% amino acid identity), followed by APS11 from *Fusarium incarnatum* (51% amino acid identity), and then a predicted MFS transporter from *Pyrenophora tritici-repentis* (46% amino acid identity). The two copies of *AjTOXA* share 95% (nucleotide) and 94% (amino acid) identity with each other. *AjTOXA* and *TOXA* each have four exons in almost the same positions (Figure 3).

**Figure 4:**
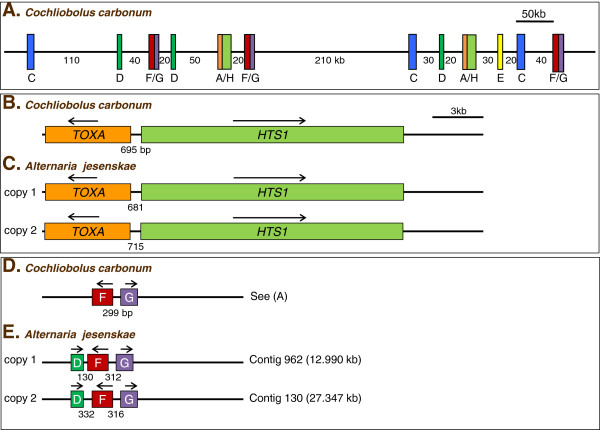
**Gene organization of the *TOX2 *genes in *C. carbonum *and *A. jesenskae*.(A)** The known organization of the *TOX2* locus in *C. carbonum* SB111 [8,9]. H = *HTS1*. **(B)** the organization of *TOXA* and *HTS1* in *C. carbonum*. **(C)** The organization of *TOXA* and *HTS1* in *A. jesenskae*. **(D)** The organization of *TOXD*, *TOXF*, and *TOXG* in *C. carbonum*. **(E)** The organization of *TOXD*, *TOXF*, and *TOXG* in *A. jesenskae*. Arrows indicate directions of transcription, except in (A) where the arrows are omitted for clarity; see ref. [9].

The two characterized copies of *AjTOXA* are clustered with the two copies of *AjHTS1*, similar to *TOXA* and *HTS1* in *C. carbonum* (Figure 4). The two genes are transcribed from opposite strands, and the predicted ATG start sites of the two genes are 681 nucleotides apart. In *C. carbonum*, the two start codons are separated by 695 nucleotides [19]. The nucleotide sequences of the four introns share 64% overall identity between the two species.

#### *AjTOXC -* fatty acid synthase beta subunit

*TOXC* in *C. carbonum* is predicted to encode a fatty acid synthase beta subunit. It is required for HC-toxin biosynthesis, probably for the biosynthesis of the decanoic acid backbone of Aeo [20]. Fungal fatty acid synthases are oligomers of alpha and beta subunits. A predicted alpha subunit gene, called *TOXH*, is clustered with the other genes of *TOX2* in *C. carbonum* but has not yet been functionally characterized (unpublished results from this lab; GenBank accession KC866372). The apicidin cluster of *F. incarnatum* and the hypothetical HC-toxin clusters of *P. tritici-repentis* and *S. turcica* (see Discussion) contain an alpha subunit gene, but, inexplicably, the clusters in neither of these two fungi, nor in *F. incarnatum*, which makes apicidin, contain a ortholog of *TOXC*[14,21,22].

There are three copies of *TOXC* in *C. carbonum*[20]. However, only one copy of *AjTOXC* was unambiguously identified in *A. jesenskae*. *AjTOXC* shares 83% (nucleotide) and 78% (amino acid) identity with *TOXC* (Table 1). *AjTOXC* has a single intron of 57 bp, and *TOXC* has a single intron of 53 bp (Figure 3). The best TBLASTN hit of *AjTOXC* in GenBank was *TOXC*.

#### *AjTOXD* (putative dehydrogenase), *AjTOXF* (putative branched-chain amino acid aminotransferase), and *AjTOXG* (alanine racemase)

BLASTN, TBLASTN, and Southern blot analyses indicated that *A. jesenskae* has at least two copies each of *TOXD*, *TOXF,* and *TOXG.* These three genes are 81-86% (nucleotide) and 81-85% (amino acid) identical to the corresponding genes in *C. carbonum* (Table 1). Gene structures were experimentally verified by sequencing 5’ and 3’ RACE products. The intron/exon structures of all *AjTOX2* genes are highly similar to *C. carbonum* (Figure 3).

These three genes are clustered together on two distinct contigs in *A. jesenskae* (Figure 4). The arrangements of the genes within each contig are different in *A. jesenskae* and *C. carbonum*. In *C. carbonum*, *TOXF* and *TOXG* are clustered within ~300 bp (Figure 4), while at least 20 kb separates *TOXD* from *TOXF* and *TOXG* in *C. carbonum*[9].

*TOXD* expression is regulated with the other genes of *TOX2* by the transcription factor TOXE, but its disruption gave no detectable HC-toxin or virulence phenotype (unpublished results from this lab). *TOXF* is required for HC-toxin production and is predicted to encode a member of the branched-chain amino acid aminotransferase family [23]. Although its precise biochemical function is unknown, a plausible function of TOXF is to aminate a precursor of Aeo, e.g., the fatty acid product of TOXC and TOXH.

The function of TOXG has been established as an alanine racemase [24]. TOXG is a member of the pyridoxal-containing serine hydroxymethyl transferase superfamily [25].

#### *AjTOXE*- HC-toxin-specific transcription factor

*TOXE* encodes a transcription factor that regulates the known genes of *TOX2* in *C. carbonum*[26,27]. It contains a bZIP DNA binding domain at its N terminus and four ankyrin repeats at its C-terminus [27]. *C. carbonum* strain SB111 has two copies of *TOXE*, one clustered with the other *TOX2* genes and one on a separate chromosome. In other strains, both copies of *TOXE* are on the same chromosome [9]. *A. jesenskae* also has two copies of *AjTOXE* on two separate contigs, but it is not known if these contigs are on the same or different chromosomes. Within *A. jesenskae* the two copies of *AjTOXE* are 85% (nucleotide) and 76% (amino acid) identical (Table 1). This is a lower degree of identity than for any of the other copies of the *AjTOX2* genes to each other. The two copies average 61% amino acid identity between *C. carbonum* and *A. jesenskae* (Table 1). This degree of conservation between TOXE and AjTOXE is lower than for any of the other TOX2 proteins (see Discussion).

In *C. carbonum*, TOXE binds to promoters of the *TOX2* genes containing the “Tox Box” motif, ATCTCNCGNA [27]. Analysis of the contigs containing the *AjTOX2* genes indicates the probable presence of similar motifs in their putative promoter regions (data not shown). However, their location in relation to the genes themselves is unclear at this time, because the transcriptional start sites of the *AjTOX2* genes have not been experimentally verified.

### Relative relatedness of housekeeping proteins and the *TOX2* proteins between *C. carbonum* and *A. jesenskae*

The percent amino acid identity of the proteins of *TOX2* and *AjTOX2* range from 58% (TOXE) to 85% (TOXF), with an average of 78.3 ± 8.3% (Table 1). In order to put this degree of relatedness in evolutionary context, we calculated the degree of amino acid identity of a set of housekeeping proteins common to most or all Dothideomycetes. The genes chosen for comparison were ones that have been characterized in *C. carbonum* and for which full-length orthologs were found in the partial *A. jesenskae* genome survey. The four housekeeping proteins ranged in identity from 76% to 96%, with an average of 84.2 ± 8.5% (Table 2). This is slightly more conserved than the *TOX2* genes, but this difference is not statistically significant.

**Table 2:** **Comparison of amino acid identities of housekeeping proteins in *C. carbonum *and *A. jesenskae***

**Protein, gene name, and GenBank accession number (in*C. carbonum*)**	**Amino acid identity (%) between*C. carbonum*and*A. jesenskae***
Cellobiohydrolase, *CEL1*, AAC49089	85
Exo-β1,3 glucanase, *EXG1*, AAC71062	76
Glyceraldehyde 3-phosphate dehydrogenase, AAD48108	96
Endo-α1,4-polygalacturonase, *PGN1*, AAA79885	76
protein kinase, *SNF1*, AAD43341	88

### Virulence of *A. jesenskae*

HC-toxin is an established virulence factor for *C. carbonum*, but any possible adaptive advantage it might confer on *A. jesenskae* is unknown. Although *A. jesenskae* was isolated from seeds of *Fumana procumbens* (it has not been isolated a second time from any source), it is not known if *A. jesenskae* is a pathogen of *F. procumbens* or any other plant. However, a number of species of *Alternaria* are plant pathogens, and specific secondary metabolites (i.e., host-selective toxins) are critical determinants of the host range and high virulence of some species and strains of this genus [3,4].

**Figure 5:**
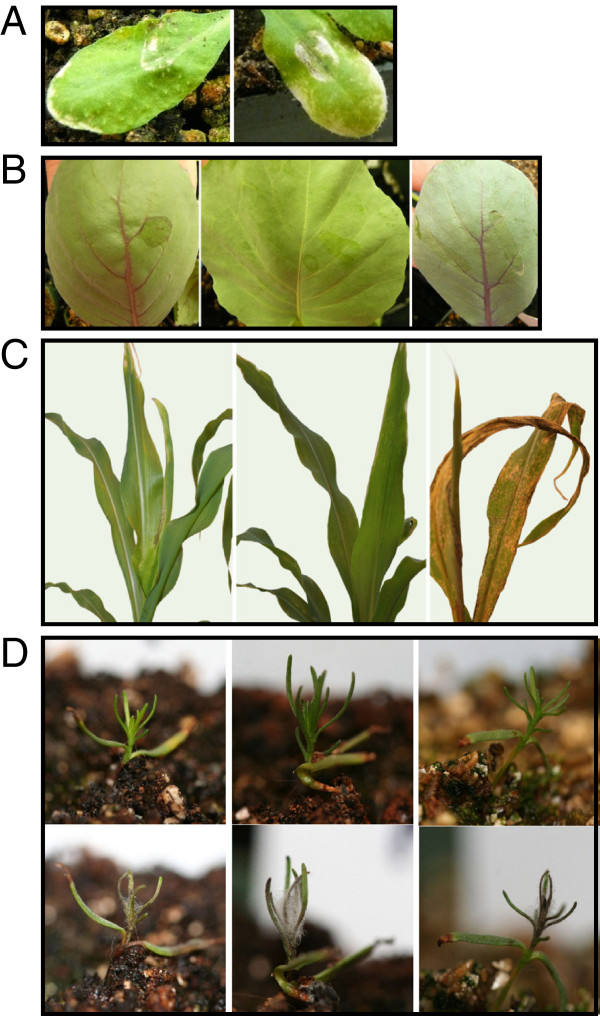
**Pathogenicity assays. (A) ***Arabidopsis thaliana* Columbia leaves 4 d after inoculation. Left panel, 0.1% Tween-20 control; right panel, inoculated with *A. jesenskae*. **(B)** Cabbage leaves 4 d after inoculation. On each leaf, 0.1% Tween alone was applied to the left side of the midvein, and *A. jesenskae* to the right side. **(C)** Left panel: maize (genotype *hm1/hm1*) inoculated with *A. jesenskae*; middle panel, maize inoculated with an isolate of *C. carbonum* that does not produce HC-toxin; right panel, maize inoculated with an isolate of *C. carbonum* that produces HC-toxin. Photographs were taken 4 d after inoculation. **(D)** Top panels, three plants of *Fumana procumbens* mock-inoculated with water; bottom panels, *F. procumbens* inoculated with *A. jesenskae*. Photographs were taken 5 d after inoculation.

In order to test whether HC-toxin has a virulence function in *A. jesenskae*, several plant species were inoculated with it. In Arabidopsis, a wild type line (Columbia), a *pad3* mutant, which has enhanced susceptibility to *Alternaria brassicicola*[28], and a quadruple DELLA mutant, which also shows enhanced susceptibility to necrotrophic pathogens such as *A. brassicicola*[29], were tested. In no case case did *A. jesenskae* cause any visible symptoms of disease (Figure 5A and data not shown). *A. jesenskae* also failed to produce any symptoms on cabbage (Figure 5B) or on maize of genotypes *hm1*/*hm1* or *HM1*/*HM1* (Figure 5C). Possible explanations for the failure of *A. jesenskae* to colonize *hm1*/*hm1* maize is that it cannot penetrate the leaves or that it does not produce HC-toxin while growing on maize. *A. jesenskae* was also tested for pathogenicity on *F. procumbens* seedlings. Under conditions of high humidity, profuse saprophytic growth was observed and most of the plants died by week 2 (Figure 5D). In some experiments, some minor symptoms of disease (i.e., chlorosis and necrosis) at inoculation sites were observed, but the symptoms did not progress and host death did not result. Therefore, we conclude that *A. jesenskae* is probably not a foliar plant pathogen.

## Discussion

This report confirms that *A. jesenskae* produces HC-toxin (R. Labuda, unpublished observations), a cyclic peptide originally found in *Cochliobolus carbonum*. A genome survey sequence of *A. jesenskae* indicated that this fungus has high-scoring orthologs of all of the known genes involved in HC-toxin biosynthesis from *C. carbonum*. The orthologs are much more closely related to each other than to any other genes or proteins in GenBank or JGI. The degree of identity makes it highly probable that these are the genes responsible for the biosynthesis of HC-toxin in *A. jesenskae*. Intron/exon structures are also highly conserved between the two fungi. It is highly unlikely that the production of HC-toxin by these two fungi evolved by convergent evolution.

In both *A. jesenskae* and *C. carbonum* the genes for HC-toxin biosynthesis are mostly duplicated and organized into a loose genomic cluster. In both fungi, the copies of *TOXA* are immediately adjacent to the two copies of *HTS1* and transcribed divergently. Some of the other genes are also clustered, but differently in the two organisms. In both fungi the multiple copies of *TOXF* and *TOXG* are tightly clustered, but whereas in *C. carbonum* all copies of *TOXD* are at least 20 kb distant from these two genes, in *A. jesenskae* both copies of *TOXD* are clustered with these two genes. Differences in gene order in clusters making the same metabolite in different fungi has been reported (e.g., ref. [30]). Further conclusions about the organization of the *AjTOX2* genes could not be deduced based on the partial genome sequence. Likewise, a full picture of the structure of *TOX2* of *C. carbonum* has not been possible due to its size, the gene duplications, and a high density of repeated elements [9].

In regard to an explanation for how two distinct species evolved the same biosynthetic machinery to synthesize the same complex secondary metabolite, there are two salient factors to consider. First, *Alternaria* and *Cochliobolus* are closely related genera in the Pleosporaceae [31]. Second, among plant pathogenic fungi, *Alternaria* and *Cochliobolus* are the major producers of known secondary metabolite host-selective toxins [1-3]. The host-selective toxins of *Alternaria* show a pattern of disjunct taxonomic distribution similar to the *Cochliobolus* host-selective toxins, i.e., production of a particular HST is typically restricted to specific strains (pathovars) or species. Compared to other groups of fungi, these two genera appear to have a particularly dynamic capacity to acquire new secondary metabolite potential, which they have successfully exploited to colonize new plant pathogenic niches.

The mechanistic basis of the generation of the extraordinary metabolic diversity in *Cochliobolus* and *Alternaria*, and more generally in the filamentous fungi, is not clear. The most plausible explanations are horizontal gene transfer and/or gene duplication followed by rapid divergence and rapid loss. Horizontal gene transfer has become increasingly accepted as an explanation for many examples of disjunct distribution of secondary metabolites and their genes. Clustering of pathway genes, a common observation, would facilitate horizontal transfer, and trans-species hyphal fusion provides a mechanism of DNA transfer [32-38]. Horizontal transfer is neither supported nor refuted by the example of HC-toxin described in this paper, because the two genera are so closely related. It is equally plausible that a common ancestor of *Alternaria* and *Cochliobolus* produced HC-toxin, and this trait was lost from most of the species in the two genera.

It is now possible to correlate genes and metabolites for three cyclic tetrapeptides of the HC-toxin family in three fungal species. *A. jesenskae* and *C. carbonum* both make HC-toxin, and their orthologous NRPS genes are 82% identical. *F. incarnatum* makes a chemically related molecule, apicidin, and its cognate NRPS (APS1) is 44% identical to HTS1. The known genes in common among the three pathways are *HTS1*, *TOXA*, *TOXC*, *TOXD*, *TOXF*, and *TOXE*. Apicidin does not contain any D amino acids besides D-proline (or D-pipecolic acid), whose production from L-proline is presumably catalyzed by the epimerase domain of APS1, and therefore an alanine racemase (TOXG) is not needed for its biosynthesis [14]. The *TOX2* cluster of *C. carbonum* contains a gene for a fatty acid synthase beta subunit (*TOXC*) and one for the alpha subunit (*TOXH*). The apicidin cluster does not contain a beta subunit gene. Either apicidin biosynthesis uses the housekeeping beta subunit, or, more likely, the gene for the dedicated beta subunit is elsewhere in the genome. The family of cyclic peptides related to HC-toxin has seven members (from seven fungi in the Sordariomycetes and Dothideomycetes) [5]. The biosynthetic genes for the other members have not yet been characterized.

Besides horizontal transfer, rapid duplication, divergence, and loss is another possible explanation for much of the observed taxonomic complexity in secondary metabolite chemistry and gene cluster structure and organization in fungi (e.g., refs. [39-41]. However, this scenario struggles to explain why secondary metabolite genes appear to have a different evolutionary trajectory than genes for primary metabolism, i.e., to what extent there are positively selected genetic mechanisms that promote diversity in secondary metabolite capacity at the expense of stability, such as transposable elements, sub-telomeric instability, and chromosomal translocations [10,22].

### Taxonomic distribution of *TOXE*

Since the discovery of this atypical transcription factor in 1998 [26], *TOXE* has been found in only a handful of other organisms, all fungi. Besides *C. carbonum* and *A. jesenskae*, reasonably strong orthologs of *TOXE* are present only in *Pyrenophora tritici-repentis*, *P. teres*, *Colletotrichum gloeosporioides*, *Setosophaeria turcica*, *Fusarium incarnatum* (*APS2*), and *Glomerella cingulata* (based on GenBank and JGI as of March, 2013). The first four fungi are in the Dothideomycetes and the second two are in the Sordariomycetes. Genes with reasonable amino acid identity and structure (i.e., containing both a bZIP DNA binding domain and ankyrin repeats) are not present in any other fungus including other species of *Cochliobolus* and *Fusarium*.

TOXE showed the lowest percent amino acid identity between *C. carbonum* and *A. jesenskae* (58-64%) of any of the TOX2 proteins, and the next best ortholog (*APS2* of *F. incarnatum*) shares only 32% amino acid identity. That these are all true orthologs can be deduced by the strong conservation of the bZIP DNA binding region at the N terminus, the ankyrin repeats at the C terminus, and by the fact that *APS2* has an experimentally determined role in regulating the biosynthesis of a secondary metabolite chemically similar to HC-toxin [14]. Apparently, the specific amino acid sequence of most of the TOXE protein is not essential for its activity. This is reminiscent of the transcription factor aflR in *Aspergillus flavus* and *A. nidulans*; the two proteins are functional orthologs despite only 33% amino acid identity [42].

*APS2* is required for expression of the apicidin biosynthetic genes [14], but the functions of the other *TOXE* orthologs are not known. In *P. tritici-repentis*, *G. cingulata*, and *S. turcica*, the *TOXE* orthologs (JGI identifiers Pyrtr1|12016, Gloci1|1721714, and Settu1|170199, respectively) are immediately adjacent to four-module NRPS genes, suggesting that the *TOXE* orthologs in these fungi have a role in regulating secondary metabolite production like they do in *C. carbonum* and *F. incarnatum*[21,22,43].

### Are there orthologs of the *TOX2* genes in other fungi?

Recently, two other fungi in the Pleosporaceae, *P. tritici-repentis* and *S. turcica*, were reported to have the *HTS1* gene [21]. This conclusion was based on the presence of a four-module NRPS clustered with genes similar to *TOXD*, *TOXA*, and *TOXE*. Putative orthologs of *TOXC*, *TOXD*, and *TOXG* were found elsewhere in the genomes of these two fungi. However, it seems unlikely that these clusters are true orthologs of *TOX2* for several reasons. First, these fungi have not been shown to make HC-toxin, and this possibility seems unlikely considering that they have been studied extensively by plant pathologists. Second, closest proximity on a phylogenetic tree does not necessarily signify that any two genes are true orthologs instead of paralogs, because in the case of taxonomically highly disjunct genes (i.e., those involved in secondary metabolism), there is no way to know how many closer orthologs actually exist among all isolates of all species in the tree. Third, the products of the individual genes of *TOX2* and the putative orthologs in *S. turcica* and *P. tritici-repentis* do not have very high amino acid identity. Orthologs of housekeeping genes in these fungi have higher amino acid identity.

A particular pitfall of assigning orthology among secondary metabolite genes whose biochemical functions are unknown is that many of them belong to broad classes of proteins that are distributed widely, being present not only in many different secondary metabolite clusters but often also having a role in primary metabolism. For example, all fungi will typically have multiple genes encoding MFS transporters (TOXA), fatty acid synthases (TOXC), short chain alcohol dehydrogenases (TOXD), and aminotransferases (TOXF). Without functional evidence, it is hazardous to attempt to associate such genes to particular secondary metabolite gene clusters within a genome.

*TOXG* (alanine racemase) serves as an example of the difficulty of identifying true orthology in fungal secondary metabolite gene clusters. The putative orthologs of *TOXG* in *P. tritici-repentis* and *S. turcica* are not clustered with the other genes of the putative HC-toxin cluster, and they are only 44% identical at the amino acid level to TOXG of *C. carbonum*. This level of identity is too low to confidently assign biochemical function, because TOXG is a member of a pyridoxal-dependent superfamily that includes enzymes with many different functions involved in both primary and secondary metabolism [25]. TOXG itself has high amino acid identity to threonine aldolase and would have been reasonably annotated as such if experimental evidence had not indicated its true function [24]. Therefore, without evidence that the putative orthologs of *TOXG* in *S. turcica* and *P. tritici-repentis* encode alanine racemases, or at least amino acid racemases, the most parsimonious interpretation is that these genes have other, unrelated functions.

The *TOX2*-like clusters in *S. turcica* and *P. tritici-repentis* probably do encode genes for the biosynthesis of cyclic tetrapeptides with at least one D amino acid (because HTS1 and its look-alikes all contain one epimerase module) and one amino acid with an aliphatic side chain (the product of TOXC, TOXH, TOXF, and other proteins). Based on the high amino acid identity among their members, the two “*TOX2*” clusters of *S. turcica* and *P. tritici-repentis* are probably true orthologs to each other, i.e., responsible for producing the same or a closely related metabolite, which has not yet been identified.

### Disjunct taxonomic distribution between species vs. disjunct distribution within species

Some secondary metabolites are present in phylogenetically disparate taxa, and others are present only in certain isolates of a single species. The distribution of HC-toxin shows both patterns: only a minority of natural isolates of *C. carbonum* produce it [6], yet, as shown in this paper, its production crosses generic boundaries. There are many documented cases of secondary metabolites being found in taxonomically unrelated species, but examples of metabolites restricted to particular isolates of a species are less common. This is probably because few fungal secondary metabolites have been studied at the population level, the host-selective toxins being an exception because of their agricultural importance and because their production is easy to score using differential plant genotypes.

Other known examples of secondary metabolites with a role in plant/pathogen interactions that are present in different genera include PM-toxin/T-toxin and fumonisins. PM-toxin and T-toxin are closely related (but not identical) linear polyketides made by *Didymella zeae-maydis* (*Phyllosticta maydis*) and *Cochliobolus heterostrophus*, respectively. Both of these fungi are in the Dothideomycetes [44]. Fumonisins are polyketide mycotoxins found in *Fusarium verticillioides* (Sordariomycetes) and *Aspergillus niger* (Eurotiomycetes). The evidence suggests that horizontal gene transfer contributed to the extant distribution of fumonisins [37].

## Conclusions

The results in this paper show that HC-toxin is made by at least one fungus outside the genus *Cochliobolus*. The genes involved in its biosynthesis are highly conserved between the two fungi. This situation could have arisen by horizontal gene transfer. Alternatively, it could have arisen by vertical transmission from a common ancestor, in which case the trait has been lost from other species of *Alternaria* and *Cochliobolus*.

## Methods

### Fungal strains and growth

*Alternaria jesenskae* was obtained from Dr. Emory Simmons (Wabash College, Crawfordsville, Indiana) and maintained on V8-juice agar plates. Its identity was confirmed by sequencing the ITS regions as described [15]. Spore suspensions were stored in 25% glycerol at -80C. Sporulation was induced by growth of unsealed plates 10 cm below a 32-watt fluorescent lamp (Philips 432T8/TL741 Universal/ Hi-Vision Hg).

### HC-toxin production and analysis

*A. jesenskae* was grown in still culture in 1-liter flasks containing 125 ml of potato dextrose broth (Difco, Franklin Lakes, NJ) for 7 to 10 d. The cultures were filtered through Whatman #1 paper and extracted twice with an equal volume of dichloromethane. The dichloromethane fractions were evaporated under vacuum at 40°C and redissolved in 3 ml methanol. After re-concentration under vacuum, the residue was dissolved in 10-50 μl of water. This crude extract was used for both TLC and HPLC. HC-toxin isolated from *C. carbonum* was used as a standard.

For TLC, extracts (10 μl) were spotted onto 250-μm silica plates with adsorbent strip (Whatman, GE Healthcare Life Sciences, Piscataway, NJ). Plates were developed in 1:1 acetone/dichloromethane. HC-toxin was detected using an epoxide-specific reagent [45]. For HPLC, 20 μl of extract was combined with 60 μl of acetonitrile and 20 μl of distilled water. The sample was injected onto a C18 reverse phase column (Eclipse XDB-C18 silica, 5 μm, 4.6 × 150 mm; Agilent, Santa Clara, CA) and was eluted with a linear gradient of 10% (v/v) acetonitrile in water to 100% acetonitrile in 30 min at a flow rate of 1 ml/min. The eluant was monitored at 230 nm. HC-toxin eluted from the column between 8 and 9 min. Mass spectrometry was performed at the MSU Mass Spectrometry Facility as described [16].

### Nucleic acid methods

DNA was extracted from 7-day old lyophilized mycelial mats of *A. jesenskae* grown in potato dextrose broth in still culture using the Gentra DNA extraction kit (Qiagen, Valencia, CA). Sequencing of genomic DNA was performed by 454 pyrosequencing at the Michigan State University Research Technology Support Facility (MSU RTSF). The total number of base pairs obtained was 483 MB. After assembly by Newbler 2.0, the number of assembled base pairs was 34.4 MB.

For DNA blotting, DNA was digested with restriction endonucleases selected specifically to evaluate gene copy number based on the genomic sequence. Internal gene-specific probes were generated based on the assembled genomic sequences. DNA was transferred to Nytran SPC (Whatman, Maidstone, England) and hybridized with ^32^P-labeled DNA probes. Specific PCR primers were used to close gaps between contigs of individual genes based on their alignment with the genes of *TOX2*.

RNA was extracted as described [46]. RT-PCR followed by 5′ and 3′ RACE was done with the SMART RACE cDNA amplification kit (Clontech, Mountain View, CA). Overlapping gene-specific primers were designed from the genomic sequence. In most cases, several gene-specific primers were used. PCR products were sequenced directly or cloned into pGem T-easy (Promega), transformed into *E. coli* DH5α (Invitrogen), and sequenced using M13 forward and reverse primers. Genomic and cDNAcopies of the genes were compared using SPIDEY (NCBI).

### Bioinformatics

BLASTN and TBLASTN searching with the genes of *C. carbonum TOX2* against the *A. jesenskae* genome used stand-alone BLAST version 2.2.15, downloaded from NCBI, and default parameters. Alignments and manual annotation of genes and proteins were done using DNASTAR Lasergene versions 7 or 8 (DNASTAR, Inc., Madison, WI), ClustalW2 (http://www.ebi.ac.uk/Tools/msa/clustalw2/), and SPIDEY (NCBI). Assembly of predicted protein sequences was performed using DNASTAR Lasergene software with assistance from FGENESH (http://www.softberry.com) with *Alternaria* as the training model. Housekeeping protein sequences were downloaded from GenBank. The *AjTOX2* genes have been deposited in GenBank with accession numbers KC862269-KC862275 (Additional file 1: Table S1).

### Virulence assays

Virulence assays on maize, cabbage, *Arabidopsis thaliana*, and *Fumana procumbens* were performed with spores collected from V8-juice plates with 0.1% Tween-20. The spore concentration was adjusted to ~10^5^ spores/ml. For maize, six- week old plants (genotype *hm1*/*hm1* or *HM1*/*HM1*) were spray-inoculated and the plants covered with plastic bags overnight to maintain humidity, after which the plants were grown in a greenhouse. Observations of disease progression were made beginning 3 d post-inoculation. For cabbage (*Brassica oleracea*), plants were grown in a growth chamber at 20°C, 70% relative humidity, and a 12-hr light /dark cycle. Leaves from 4-week-old plants were spot-inoculated with 10 μl of inoculum. Plants were covered overnight to maintain humidity. Plants were observed for signs of infection beginning 4 d after inoculation. For Arabidopsis, plants (Col-0, a *pad3* near-isogenic mutant, and a DELLA quadruple mutant [29]) were grown in a growth chamber at 20°C, 70% relative humidity, and a 12-hr light/dark cycle. The third through the seventh true leaves from 4-week-old plants were spot-inoculated with 10 μl of spores. Plants were covered overnight to maintain humidity and observed for signs of infection starting 4 d after inoculation.

Seeds of *Fumana procumbens* were obtained from Hardyplants, Apple Valley, MN, and after scarification with a razor blade were germinated in glass scintillation vials on Whatman #1 filter paper. Seven to ten day-old seedlings were transferred to soil and grown at room temperature under a 32 watt fluorescent light (Philips 432T8/TL741 Universal/ Hi-Vision Hg). Conidial suspensions of *A. jesenskae* (10 μl) were applied as a drop on the surface of leaves of 5-6 month old plants. Plants were covered with a clear plastic dome lid and kept at 100% relative humidity for 48 hr. Observations were made beginning 3 d after inoculation.

## Competing interests

The authors declare that they have no competing interests.

## Authors’ contributions

WW did most of the experimental work and wrote the first draft of the manuscript. RL discovered that *A. jesenskae* makes HC-toxin. JW did some of the bioinformatics analysis and wrote the final draft of the manuscript. All authors read and approved the final manuscript.

## Supplementary Material

Additional file 1 **Conservation of the genes for HC-toxin biosynthesis in *Alternaria jesenskae*. Table S1.** GenBank accession numbers for genes of *TOX2* and *AjTOX2*. **Table S2.** List of primers used to amplify probes used for Southern blots (Figure 2). Click here for file
