# Supplementary material for: Conservation of the genes for HC-toxin biosynthesis in Alternaria jesenskae
Source: BMC Microbiol. 2013 Jul 17;13:165. doi: 10.1186/1471-2180-13-165 (PMC3729494; doi:10.1186/1471-2180-13-165)
Supplement: Additional file 1 — Conservation of the genes for HC-toxin biosynthesis in Alternaria jesenskae. Table S1. GenBank accession numbers for genes of TOX2 and AjTOX2. Table S2. List of primers used to amplify probes used for Southern blots (Figure 2). [file 1471-2180-13-165-S1.docx]

**Supplementary Material**

Wight, Labuda and Walton “**Conservation of the genes for HC-toxin biosynthesis in *Alternaria* *jesenskae*”**

**Table S1. GenBank accession numbers for genes of *TOX2* and *AjTOX2*.**

| Gene | *Cochliobolus carbonum* | *Alternaria jesenskae* |
| --- | --- | --- |
| *HTS1* | M98024.2 | KC862269 |
| *TOXA* | L48797.1 | KC862270 |
| *TOXC* | U73650.1 | KC862271 |
| *TOXD* | X92391.1 | KC862272 |
| *TOXE* | AF038874.1 | KC862273 |
| *TOXF* | AF157629.1 | KC862274 |
| *TOXG* | AF169478.1 | KC862275 |

**Table S2. List of primers used to amplify probes used for Southern blots (Figure 2).**

| Name | Forward Primer | Reverse Primer |
| --- | --- | --- |
| *AjHTS1* | GAGATTCTGTACACAGGGGTAGGAGGT | TCTTTGCCCCTCACCACTAACAATGCCGTT |
| *AjTOXA* | TTGCTCATGAGCGGAACTATGCAGCCCCTT | AAAGCTCCGATCATACCAGTGAAGGTGGCT |
| *AjTOXC* | GCTGGCTTGAGGAGTATTGG | AATGTGAGTTGCTCCAGG |
| *AjTOXD* | TGAAAACTCAAGTTCTCTGGTATCCATGCCTGA | GCGACTTCATGAGCTCAAAATTGTGGGGAG |
| *AjTOXF* | CTTGCGGGCACTGATAGCGGAGAAATTTGA | GCTCATCCTCTTCTCCGGCCTTTTTCATTC |
| *AjTOXG* | CATGCTATACACTATGAGGCAGGTGGCCCT | AGTGTGTCTTGACGTTGTTTTCCTTTGCCC |
